# DArTseq molecular markers associated with water stress tolerance and size reduction in citrandarins rootstocks

**DOI:** 10.3389/fpls.2026.1857861

**Published:** 2026-08-10

**Authors:** Fernanda Roverssi, Luciane Santini-Gazaffi, Ana Júlia Borim de Souza, Fernando Trevizan Devite, Gustavo Henrique Colombo, Thiago Peitl Monteiro, Fernando Alves de Azevedo, Mariângela Cristofani-Yaly

**Affiliations:** Sylvio Moreira Citrus Center, Agronomic Institute of Campinas, Cordeirópolis, São Paulo, Brazil

**Keywords:** blast, citrandarins, drought tolerance, *Poncirus trifoliata* cv. Rubidoux, SilicoDArT

## Abstract

In Brazil, most orchards are not irrigated and flowering usually occurs in August/September, the season with the least rainfall, making it necessary to use drought-tolerant rootstocks. Reduced scion vigor, associated with drought tolerance, are essential factors for high-density planting systems in conditions of limited water availability. The identification of molecular markers potentially associated with these characteristics is essential to accelerate the improvement of citrus rootstocks. This study aimed to identify DArTseq molecular markers related to dwarfism and drought tolerance in citrandarin rootstocks (*Citrus sunki* × *Poncirus trifoliata*) using an *in silico* Bulk Segregant Analysis (BSA) approach. Experiments were conducted in Barretos, a municipality in the northern region of the São Paulo state. The bulks were selected based on vegetative development and leaf rolling scores for drought tolerance. The molecular markers were identified through differences in allele frequency between the groups, and the markers putatively associated underwent functional annotation to infer candidate genes for these characteristics using the Blast2GO program with the NCBI nr database. A total of 12 candidate markers were identified and associated with the characteristics of interest, including six related to reduced vigor and six to drought tolerance. The functional annotation enabled the identification for gene sequences associated with biological processes related to plant development, root architecture, hormonal regulation, and response to abiotic stress. The identified markers represent candidate genomic regions potentially involved in vigor regulation and drought response. These results represent valuable resources for future research in citrus rootstock breeding programs.

## 1 Introduction

Brazil stands out as the world’s leading producer of oranges and exporter of orange juice. In 2024, it produced approximately 15.7 million tons of fruit from a harvested area of 565 thousand hectares. The state of São Paulo is the main citrus-producing region in Brazil, with approximately 12 million tons of fruit, corresponding to 77% of the national orange production (IBGE, 2026). Despite its great importance, commercial orchards rely on a limited number of varieties, which represents a high phytosanitary risk, therefore, diversification of the production base is essential to prevent problems already experienced by Brazilian citriculture (Oliveira et al., 2014).

In addition to the limited genetic base, environmental factors also pose challenges to production. In Brazil, most citrus orchards are not irrigated, and flowering generally occurs from August to September, a period of lower rainfall. This condition makes the use of drought-tolerant rootstocks and/or those more efficient under water restriction necessary. Given these conditions, the choice of rootstock becomes a critical factor in citriculture (Pompeu Junior, 2005).

Certain rootstocks can reduce canopy size by decreasing tree vigor (Donadio et al., 2019). Small citrus trees exhibit greater productive efficiency, allow higher planting densities, and consequently enable greater production per unit area. In addition, smaller tree size facilitates harvesting, inspection, spraying, and plant eradication in the control of HLB (Stuchi et al., 2012).

The reduction in scion development when grafted onto dwarfing rootstocks is associated with lower water potentials in leaves and stems compared to plants grafted onto vigorous rootstocks (Martínez-Cuenca et al., 2016). Dwarfing rootstocks have smaller root systems and, consequently, their roots absorb less water and nutrients from the soil (Hayat et al., 2022). Consequently, water transport within the plant may be restricted, leading to greater water deficit in dwarfing plants.

The rootstock *Poncirus trifoliata* var. *monstrosa* cv. ‘Flying Dragon’ is a widely used commercial dwarfing rootstock, however, it presents serious incompatibility issues with some scion cultivars and is susceptible to drought-induced water stress (Stuchi et al., 2003). Other dwarfing rootstocks derived from hybrids of trifoliate orange exist which represent alternatives for orchards under high-density planting systems (Gonzatto et al., 2022).

Hybrid selections have been developed in breeding programs in Spain (Martínez-Cuenca et al., 2016) and in breeding programs conducted in Brazil by the Agronomic Institute (IAC) (Schinor et al., 2013) and by Embrapa (Ramos et al., 2015). The Sylvio Moreira Citrus Center/IAC has been conducting a citrus genetic breeding program based on controlled crosses since 1990. Populations of citrandarin hybrids (*C. sunki* × *P. trifoliata* cv. Rubidoux), selected using molecular markers, were established in different regions of the state of São Paulo. Some of these hybrids showed drought tolerance comparable to that of Rangpur lime and also conferred different plant sizes to the scion variety (Cristofani-Yaly et al., 2007; Schinor et al., 2013).

Recent studies indicate high potential in several IAC citrandarins. The citrandarin IAC 3152 Itajobi (H152) showed remarkable resilience to water stress, outperforming ‘Flying Dragon’ in water potential and photosynthetic efficiency (De Souza et al., 2025). The citrandarins Changsha × English Large (IAC 1711) and the citranges Troyer (IAC 385) and Carrizo (IAC 387) also show potential as rootstocks under rainfed conditions (Silva et al., 2025). Additionally, the citrandarin IAC 1600 proved to be more adapted to regions with limited water availability and high incidence of HLB (Devite et al., 2025). These studies highlight the importance of developing new rootstock varieties and improving the characterization of existing ones.

In this regard, biotechnology has supported citrus breeding programs through controlled hybridization and the use of molecular markers in studies of genetic diversity, identification of zygotic and nucellar seedlings, genetic mapping, and marker-assisted selection (MAS) (Curtolo et al., 2017; Lima et al., 2018).

In this context, high-throughput genotyping technologies stand out, such as the DArTseq™ platform (Diversity Arrays Technology–DArT), which combines genome complexity reduction methods with modern sequencing technologies, generating thousands of dominant markers distributed across the genome. In citrus, DArTseq markers have been used to construct highly dense genetic linkage maps for *Citrus sunki* and *Poncirus trifoliata*, contributing to the genetic improvement of the crop (Curtolo et al., 2018).

Another relevant technique is Bulked Segregant Analysis (BSA) (Michelmore et al., 1991), which compares pools of individuals with contrasting phenotypes within a segregating population and genotyped with molecular markers that are polymorphic between the parents. With the emergence of next-generation sequencing (NGS) technologies, BSA approaches have drastically accelerated the process of gene identification (Schneeberger, 2014). In citrus, the BSA technique has been used to map a resistance locus for citrus nematode and for Alternaria brown spot (Xu et al., 2010; Cuenca et al., 2013).

The inheritance of the dwarfing trait was studied in a Flying Dragon progeny, in which the characteristic was determined by a dominant gene. In this study, BSA was used to identify three RAPD molecular markers linked to the dwarfing gene (Cheng and Roose, 1995).

These approaches, including the use of high-density molecular markers (such as DArTseq) and BSA-based strategies, have been successfully used to identify candidate genes for important traits. Song et al. (2017) used NGS and BSA to simultaneously detect two genes (*qCC1* and *qCC2*) controlling cotyledon color in soybean. This method allows loci to be rapidly associated with candidate genomic regions.

New methods have been developed to accelerate and optimize the identification of genomic loci, such as QTL-seq (BSA QTL-seq), which uses genotype-guided bulk reconstruction through bioinformatics, allowing the simultaneous mapping of multiple traits from the same genotypic dataset (Esposito et al., 2026). *In silico* bulked segregant analysis (*in silico* BSA) is a computational approach that mimics traditional bulked segregant analysis using existing genotypic and phenotypic data, without the need to physically construct DNA bulks. In this method, individuals from a segregating population are virtually grouped (bulked) based on their phenotypes (e.g., extreme traits), and allele frequency differences between these digital bulks are analyzed using sequencing or marker data. By comparing these groups, researchers can identify genomic regions associated with traits of interest (QTLs) (Bello et al., 2014).

The association between genes and phenotype represents an important step toward understanding the mechanisms involved in these traits. In this context, the breeding program genotyped the hybrid population of *C. sunki* × *P. trifoliata* and obtained genetic linkage maps with DArTseq markers and SNPs for *P. trifoliata* and *C. sunki* through Diversity Arrays Technology Pty Ltd (DArT P/L, Canberra, Australia) (Curtolo et al., 2018). This same population was recently used to construct a high-density, multiplatform map and to identify HLB response QTLs in several trials (Santini-Gazaffi et al., 2026).

Despite the progress achieved in the development of new citrus rootstocks, the genetic architecture underlying drought tolerance and dwarfing remains poorly understood. Previous studies have characterized agronomic performance and stress responses of several citrandarin genotypes, and high-density genetic maps are available for the *Citrus sunki × Poncirus trifoliata* population. However, genomic regions associated with drought tolerance and canopy size control have not yet been systematically identified in this population using integrated phenotypic and genome-wide marker information. Furthermore, the application of *in silico* bulked segregant analysis (BSA) combined with DArTseq-derived markers for the dissection of these traits in citrus rootstocks remains largely unexplored.

The novelty of the present study lies in the integration of long-term field phenotyping data with high-density DArTseq genotyping and *in silico* BSA to identify genomic regions associated with drought tolerance and dwarfing in citrandarin rootstocks. To our knowledge, this is the first study to apply this combined approach in a segregating *C. sunki × P. trifoliata* population under field conditions, providing new insights into the genetic basis of two key traits for the development of resilient and high-density citrus production systems.

Therefore, the objective of this study was to identify genomic regions associated with drought tolerance and dwarfing in citrandarin rootstocks derived from *C. sunki × P. trifoliata* through the integration of field phenotypic evaluations, high-density molecular markers, and *in silico* bulked segregant analysis.

## 2 Materials and methods

### 2.1 Experimental area

The experiment was established in 2013 in a municipality in the state of São Paulo, Brazil: Barretos (Northern Region/SP – Guanabara Farm, latitude 20°30’32.30” South and longitude 48°36’33.99” West, 536 m altitude, soil classified as Dystroferric Red Latosol, Aw climate, tropical).

Experiments were established with ‘Pera’ sweet orange grafted onto 20 rootstocks, including 17 citrandarins of *C. sunki* × *P. trifoliata* cv. Rubidoux and citrandarin IAC 1697 (*C. sunki* × *P. trifoliata* cv. Benecke). The Swingle citrumelo (SW) [*C. paradisi* Macfad. × *P. trifoliata* (L.) Raf.] and citrumelo W2 were evaluated as standard rootstock varieties (**Table 1**).

**Table 1:** Rootstocks evaluated in the experiment, parents, commercial name, and registration number in the MAPA registry.

Abbreviation	Parents	Commercial name	RNC/MAPA
H18	*C. sunki* x *P. trifoliata* cv. Rubidoux		–
H26		IAC 3026 Santamélia	44688
H47			–
H68			–
H70			–
H73			–
H110			–
H124			–
H128		IAC 3128 Guanabara	44689
H137			–
H139			–
H148			–
H150			–
H151			–
H152		IAC 3152 Itajobi	36027
H248			–
H299		IAC 3299 Muriti	36025
IAC 1697	*C. sunki* x *P. trifoliata* cv. Benecke	IAC 1697	21832
SW	*C. paradisi* Macfad. x *P. trifoliata* (L.) Raf.	Citrumelo Swingle	–
W2		Citrumelo Swingle W2	–

In both experiments, the experimental design was a randomized complete block with three replications and a variable number of plants per plot, spaced at 6.5 × 2.8 m (550 trees per hectare). For phenotypic evaluations, one representative plant per plot was used, totaling three biological replicates per genotype.

Plants were cultivated following the standard practices recommended for sweet orange production in Brazil, without pruning or fruit thinning. Weeds were controlled by mechanical mowing of natural vegetation (*Brachiaria* spp.) between rows, supplemented with herbicide applications along the planting rows. The experiment has been conducted under irrigation.

**Figure 1:**
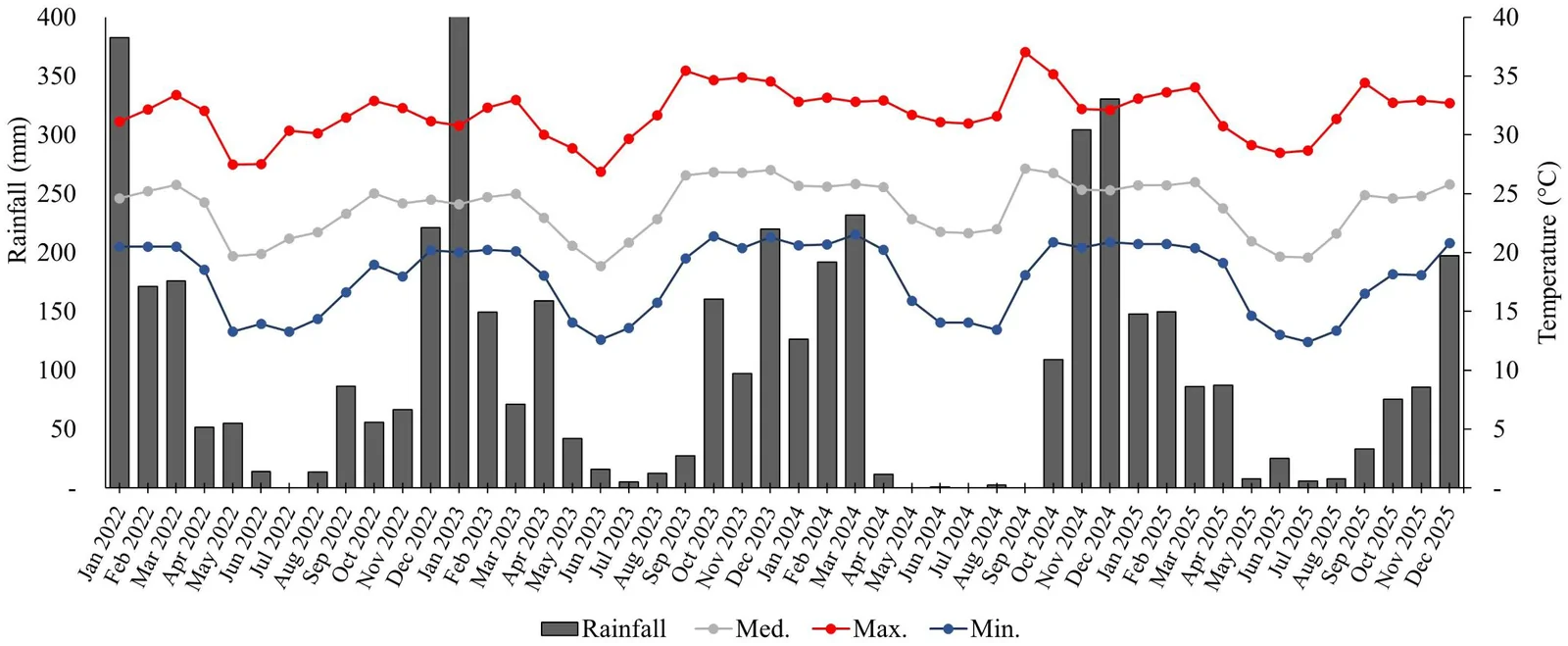
Precipitation and maximum, mean, and minimum temperatures in Northern region of the state of São Paulo. 2022-2025.

Environmental conditions were monitored, including precipitation and temperature (maximum, mean, and minimum), allowing characterization of contrasting water availability between years 2022 to 2025 (**Figure 1**). In Barretos (northern region of São Paulo), where July, August, and June had the lowest precipitation. Overall, precipitation was lower in 2024 compared to others years.

Based on the climatic data from northern region was slightly warmer and had lower precipitation levels, with March being the warmest month and May the coldest. These seasonal variations allowed the evaluation of plant performance under natural conditions of water restriction (deficit conditions), particularly during winter months.

### 2.2 Vegetative development

Plant development, represented by canopy volume (m³), was measured in 2022, 2023, 2024, and 2025 in three replications, with one plant per plot. The variable was determined using a graduated ruler by measuring plant height in meters (H) and canopy diameter (D), measured parallel to the ground at a height of 1.5 meters. Subsequently, canopy volume (V) was calculated using the following equation:


$$V=\frac{2}{3}\times \pi \times {\left(\text{R}\right)}^{2}\times H$$

(Mendel, 1956).

### 2.3 Drought tolerance

A visual assessment of drought tolerance was performed in 2022, 2024 and 2025 (winter). These assessments consisted of assigning visual scores based on the presence and/or absence of leaf curling characteristic of plants under water stress, with three repetitions per treatment, as used by Stuchi et al. (2000). Scores ranging from 1 to 3 were assigned, where: (1) significant leaf rolling, with or without a dry appearance; (2) slight leaf rolling, leaves slightly rolling; and (3) normal leaf blade, absence of leaf curling. For both analyses, three repetitions were used, one plant per plot, totaling three biological repetitions per genotype.

### 2.4 Statistical analysis

All data were analyzed using analysis of variance (ANOVA) and the Scott-Knott mean comparison test with a significance level of α = 0.05. When necessary, a Box-Cox transformation (Box and Cox, 1964) was applied to normalize the data distribution. The data analysis was conducted using R statistical software (version 4.4.2; Foundation for Statistical Computing, Vienna, Austria) (R Core Team, 2024), utilizing the ExpDes.pt package (Ferreira et al., 2021) and the MASS package (Venables et al., 2002).

### 2.5 Selection of molecular markers

The search for genomic regions associated with dwarfing and drought tolerance traits was conducted using the Bulk Segregant Analysis (BSA) *in silico* approach (Bello et al., 2014). The hibrids are part of a breeding program involving the segregating population derived from *C. sunki × P. trifoliata*, previously genotyped using DArTseq markers (Curtolo et al., 2018), enabling the integration of phenotypic and molecular data. The DArTseq markers used were coded as 0 (absent) or 1 (present).

Plant selection for the construction of bulks related to induced tree size was performed using statistical data for the following traits: canopy height (m), canopy diameter (m), and canopy volume (m³). The selection considered groups that showed statistically significant differences according to the Scott–Knott test (α = 0.05).

For the selection of bulks associated with drought tolerance, the visual leaf curling score obtained in 2024 was used. Similarly, the selection of tolerant/intolerant bulks was performed considering groups with statistically significant differences according to the Scott–Knott test (p > 0.05), with group A classified as tolerant and group B as intolerant.

DArTseq markers association with phenotypic data was assessed by grouping the 17 evaluated hybrids into two bulks (A and B). These bulks were formed from groups with extreme values identified through analysis of variance and the Scott-Knott test for each evaluated trait. We calculated allele frequency (presence/absence) for each locus and determined the contrast between the frequencies of each bulk using the $\Delta Allele_{index}$. This index, adapted from Takagi et al. (2013), is calculated as follows: $\Delta Allele_{index}=Allele\;frequency_{Bulk\;A}-Allele\;frequency_{Bulk\;B}$. The statistical significance of the ΔAllele index was assessed using Fisher’s exact test (Fisher, 1922), with a significance level α = 0.05.

The selection of markers associated with plant size employed a conservative approach, focusing only on those markers associated with all the evaluated traits: height, diameter, and canopy volume. A Venn diagram (https://bioinformatics.psb.ugent.be/webtools/Venn/) was constructed using the molecular markers significantly associated with each trait, and only those corresponding to the intersection of the datasets were selected for subsequent analyses.

### 2.6 Annotation of sequences in *Citrus* genomes

After selecting DArTseq markers through *in silico* BSA, the marker sequences were aligned using BLASTn algorithm (Basic Local Alignment Search Tool) against the *Citrus sinensis* reference genome (RefSeq ID GCF_022201045.2) available at the NCBI (National Center for Biotechnology Information) nonredundant (nr) database (http://www.ncbi.nlm.nih.gov). Gene sequences located closest to the selected markers were annotated using the Blast2GO software v. 6.0.3 (Conesa et al., 2005).

Similarity searches were performed using BLASTx against the NCBI nr database, applying a taxonomic filter for Viridiplantae (green plants) and an e-value cutoff of 1×10^−5^. Subsequently, the sequences were analyzed using InterProScan to identify conserved domains, followed by Gene Ontology (GO) term mapping and functional annotation based on the obtained GO candidates.

## 3 Results and discussion

### 3.1 Vegetative development

Regarding tree height, ‘Pera’ sweet orange grafted onto H150, H248, H70, H151, H139, H124, and H18 did not differ from W2, showing greater plant height (≥3.5 m). For canopy volume, plants grafted onto H18, H124, H70, SB, H248, W2, H128, H139, and H151 did not differ from W2, exhibiting greater canopy volume (>30 m³).

In contrast, plants grafted onto H148, H110, H73, H68, H137, and H26 developed smaller trees, with heights up to 2.8 m. Plants grafted onto H110, H68, H137, and H26 induced lower canopy volume (≤20 m³). The rootstocks H18, H124, SB, H70, H128, W2, and H248 induced greater canopy diameter (**Table 2**).

**Table 2:** Height, canopy diameter, and canopy volume of ‘Pera’ sweet orange grafted onto 20 rootstocks in Barretos, (Northern Region) São Paulo State, Brazil, 2025.

Rootstock	Height (m)	Diameter (m)	Canopy volume (m^3^)
H18	3,50a	4,73a	41,45a
H26	2,17f	3,67b	15,27c
H47	3,00c	4,07b	26,02b
H68	2,63e	3,67b	18,56c
H70	3,70a	4,33a	36,37a
H73	2,80d	3,93b	22,67b
H110	2,83d	3,73b	20,70c
H124	3,53a	4,47a	36,92a
H128	3,40b	4,27a	32,73a
H137	2,53e	3,60b	17,25c
H139	3,57a	4,07b	30,88a
H148	2,87d	3,87b	22,42b
H150	3,73a	3,67b	26,75b
H151	3,63a	4,00b	30,69a
H152	3,30b	3,80b	25,19b
H248	3,70a	4,13a	33,68a
H299	3,10c	4,00b	26,01b
IAC 1697	3,37b	4,47a	35,54a
SW	3,20b	4,07b	27,73b
W2	3,60a	4,20a	33,27a
CV (%)	4,73	7,46	16,75

Means followed by the same letter among rootstocks do not differ from each other according to the Scott–Knott test (p > 0.05).

Studies involving *P. trifoliata* rootstock and some of its hybrids have demonstrated their ability to reduce the height and canopy volume of ‘Valencia’ sweet orange under rainfed conditions (Domingues et al., 2021). According to Pompeu Junior (2005), a dwarfing rootstock is defined as one that results in a mature tree with a height of less than 2.5 m, regardless of the influence of climate, soil, or irrigation.

The SW citrumelo is a vigorous rootstock that induced greater canopy growth in ‘Valencia’ sweet orange (Devite et al., 2025). According to Pompeu Junior and Blumer (2011), both SW and W2 rootstocks produced larger and more productive orange trees, with the same production efficiency as those grafted onto Rangpur lime. Therefore, it can be inferred that H18 and H128 are vigorous rootstock varieties, as they did not differ from the standard SW and W2 varieties and exhibited greater vigor regardless of location.

### 3.2 Drought tolerance

**Figure 2:**
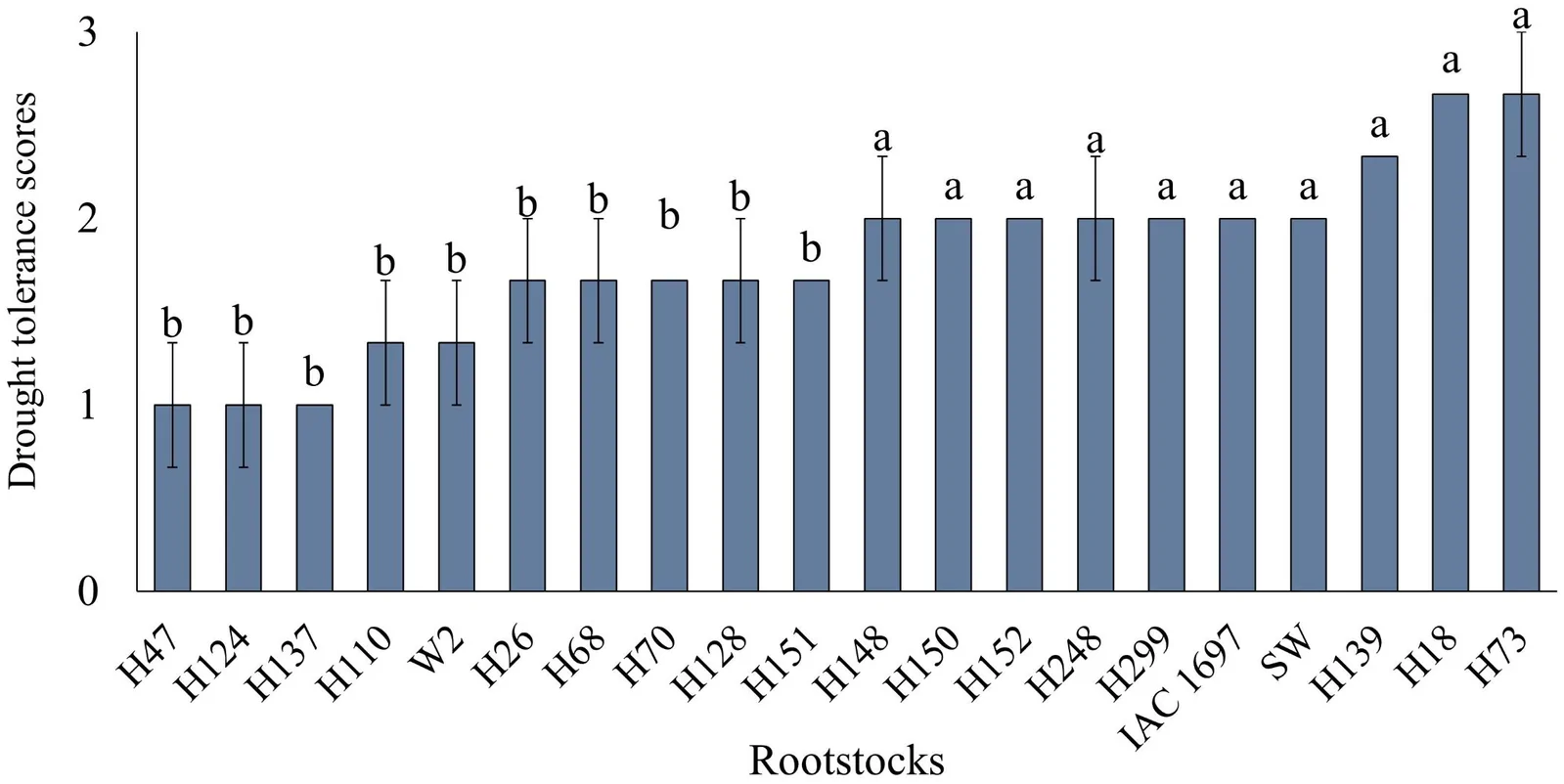
Leaf rolling scores in in ‘Pera’ sweet orange in Barretos, São Paulo State, Brazil, 2024. Means followed by the same letter do not differ according to the Scott–Knott test (pvalue ≤ 0.05).

As observed in the climatic data presented in **Figure 1**, the northern region of São Paulo was under greater water deficit, particularly in 2024. In general, precipitation was lower in 2024 compared to others years. (**Figure 1**), which is reflected in leaf rolling scores values observed under these conditions (**Figure 2**).

Since June 2023, the citrus belt has been affected by the El Niño phenomenon, one of the five most intense events ever recorded. The combination of high temperatures, elevated evapotranspiration rates, and severe water deficit across the region resulted in a reduced number of fruits per tree (FUNDECITRUS, 2024).

In ‘Pera’ sweet orange grafted onto the hybrids H18, H73, H139, H148, H150, H152, H248, H299 and SW exhibited greater drought tolerance based on visual assessment in 2024 (**Table 3**). These rootstocks received higher drought tolerance scores, reflecting lower levels of leaf curling. In contrast, H128, H151, H26, H68, H70, H110, W2, H124, H137 and H47 exhibited the lowest scores.

**Table 3:** Drought tolerance in ‘Pera’ sweet orange grafted onto different rootstocks (RS), evaluated based on leaf curling score in Barretos, São Paulo State, Brazil.

Rootstock	2022	2024	2025	Média
H18	1,33b	2,67a	2,33a	2,11a
H26	1,33b	1,67b	2,33a	1,78a
H47	2,00a	1,00b	2,00a	1,67a
H68	2,00a	1,67b	1,33a	1,67a
H70	2,33a	1,67b	2,00a	2,00a
H73	1,33b	2,67a	2,00a	2,00a
H110	2,67a	1,33b	2,33a	2,11a
H124	2,33a	1,00b	2,67a	2,00a
H128	1,67b	1,67b	2,00a	1,78a
H137	2,33a	1,00b	2,67a	2,00a
H139	2,00a	2,33a	1,67a	2,00a
H148	2,00a	2,00a	2,67a	2,22a
H150	2,00a	2,00a	2,33a	2,11a
H151	1,67b	1,67b	2,00a	1,78a
H152	2,33a	2,00a	2,00a	2,11a
H248	2,00a	2,00a	2,00a	2,00a
H299	2,00a	2,00a	2,00a	2,00a
IAC 1697	2,00a	2,00a	2,00a	2,00a
SW	1,67b	2,00a	2,00a	1,89a
W2	2,00a	1,33b	2,00a	1,78a
CV%	23,01			

Means followed by the same letter among rootstocks do not differ from each other according to the Scott–Knott test (p > 0.05).

According to Schinor et al. (2013), scores ranging from 1.0 to 1.3 indicate high susceptibility, scores from 1.5 to 2.1 indicate moderate susceptibility, whereas scores between 2.2 and 3.0 indicate drought tolerance. In the same study, the citrandarins H152, H248, and H299 also exhibited high drought tolerance when used with ‘Pera’ sweet orange.

The female parent, *C. sunki*, was considered more tolerant, inducing higher drought tolerance scores in ‘Folha Murcha’ sweet orange. In contrast, the rootstock *P. trifoliata* ‘Flying Dragon’ conferred the lowest level of drought tolerance (Cantuarias-Avilés et al., 2011). In Valencia sweet orange, Sunki mandarin and some of its hybrids induced greater drought tolerance (Stuchi et al., 2000; Pereira Costa et al., 2021).

### 3.3 Selection of DArTseq molecular markers

For the trait of drought stress tolerance, the hybrids H18, H73, H139, H148, H150, H152, H248, and H299 exhibited higher tolerance values, whereas H26, H68, H70, H128, H151, H110, H47, H124, and H137 showed lower values. These were selected to compose the tolerant and susceptible bulks, respectively. Among 27,960 DArTseq markers, it was possible to identify 12 markers through *in silico* Bulk Segregant Analysis (BSA) using Fisher’s exact test (**Supplementary Table 1**).

**Figure 3:**
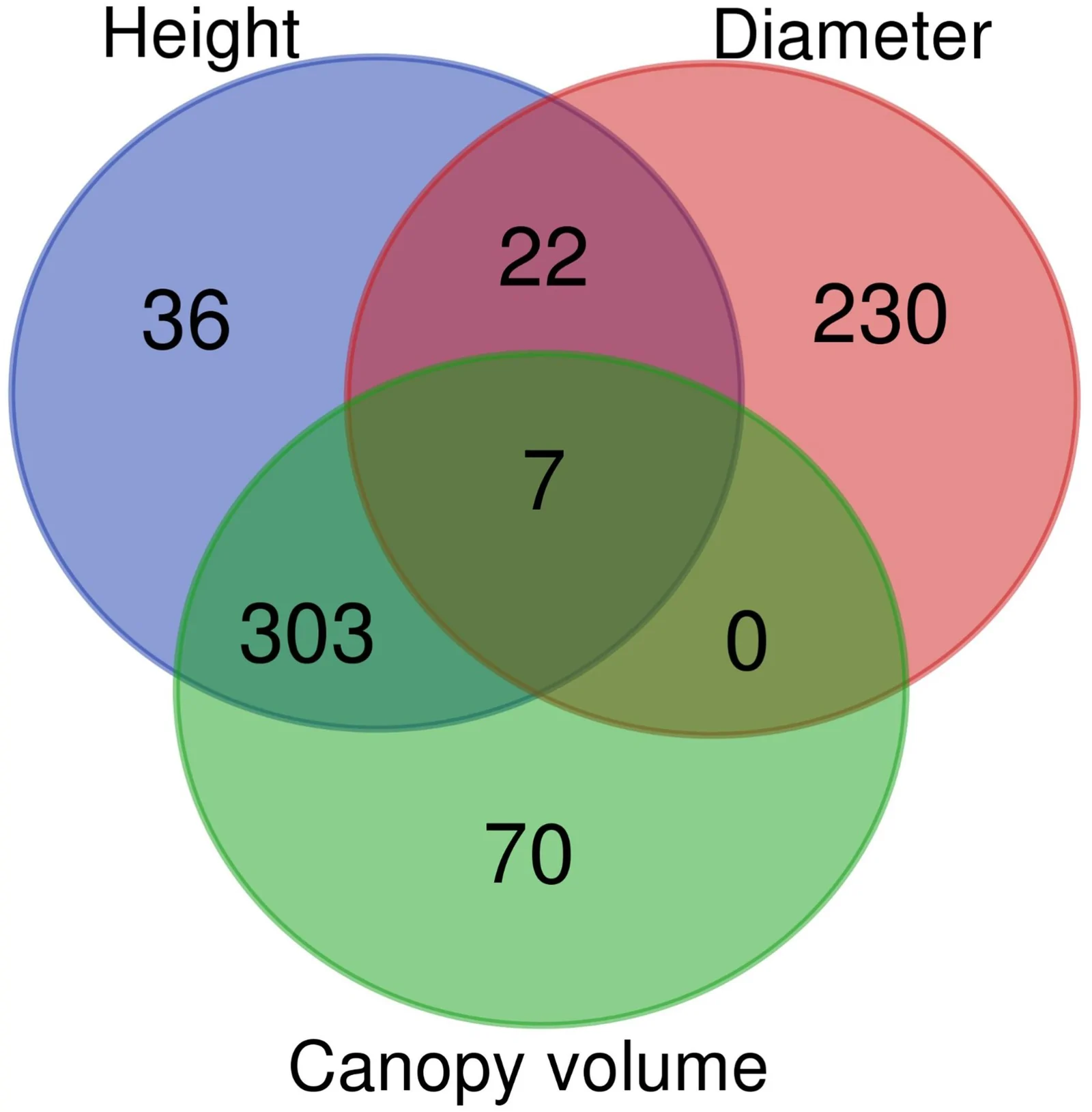
Venn diagram of DArTseq molecular markers associated with height, diameter and canopy volume induced.

Regarding the canopy size induced by the rootstocks, significant markers were initially selected using Fisher’s exact test for the individual traits: tree height, canopy diameter, and canopy volume (**Supplementary Table 1**). Marker selection identified 368 markers putatively related with tree height, 259 with canopy diameter, and 380 with canopy volume. Among these markers, seven (7) were common to all three traits, as determined using a Venn diagram (**Figure 3**).

### 3.4 Functional annotation

Among the 12 significant molecular marker sequences putatively related with drought tolerance, seven aligned with gene sequences in the NCBI database of *C. sinensis*, and all sequences showed identity ≥ 89% (**Table 4**). Marker 100044107|F|0 presented multiple copies in the genome and was therefore not considered specific and was not used for annotation.

**Table 4:** DArTseq molecular markers aligned to the *Citrus sinensis* reference genome (assembly DVS_A1.0) available at the NCBI database.

DArTseq	Chromosome location	Hit location	Direction	Score	Identity %	Gene ID
Putatively associated with drought tolerance						
100026369|F|0	Chr3 NC_068558.1	10.666M	forward	84	97	LOC112498199
100019255|F|0	Chr4 NC_068559.1	26.612M	reverse	129	100	LOC102621883
100010748|F|0	Chr9 NC_068564.1	7,550.7K	reverse	86	100	LOC102610219
100023347|F|0	Chr2 NC_068557.1	3,400.5K	reverse	97	92	LOC112497242
100046832|F|0	Chr7 NC_068562.1	1,231.9K	reverse	117	97	LOC107178890
100044107|F|0	Chr1 NC_068564.1	2,997.8K	forward	75	95	KPL70_000629
100044107|F|0	Chr9 NC_068564.1	6,987,0K	forward	53	100	LOC102617533
100023207|F|0	Chr9 NC_068564.1	31,002M	reverse	90	89	KPL70_027387
Putatively associated with dwarfing vigor						
100033871|F|0	Chr2 NC_068557.1	25.234M	forward	77	100	LOC127900450
100009554|F|0	Chr3 NC_068558.1	31.646M	reverse	81	90	LOC127898710
100010962|F|0	Chr3 NC_068558.1	28.886M	reverse	129	100	KPL70_007989
100010962|F|0	Chr3 NC_068558.1	28.991M	reverse	119	98	LOC112496823
100010962|F|0	Chr3 NC_068558.1	28.487M	reverse	106	94	LOC107175105
100010962|F|0	Chr3 NC_068558.1	28.672M	reverse	95	91	LOC107175243
100048650|F|0	Chr3 NC_068558.1	16.190M	forward	53	100	LOC102619505
100053083|F|0	Chr2 NC_068557.1	25.234M	forward	77	100	LOC127900450
100021565|F|0	Chr3 NC_068558.1	41.749M	reverse	103	95	KPL70_009179

Among the seven significant molecular marker sequences putatively related with dwarfing traits, six aligned with gene sequences in the NCBI database of *C. sinensis*, and all sequences showed identity ≥ 91% (**Table 4**). Marker 100010962|F|0 aligned at more than one genomic position in *C. sinensis*; only sequences containing genes were annotated.

**Figure 4:**
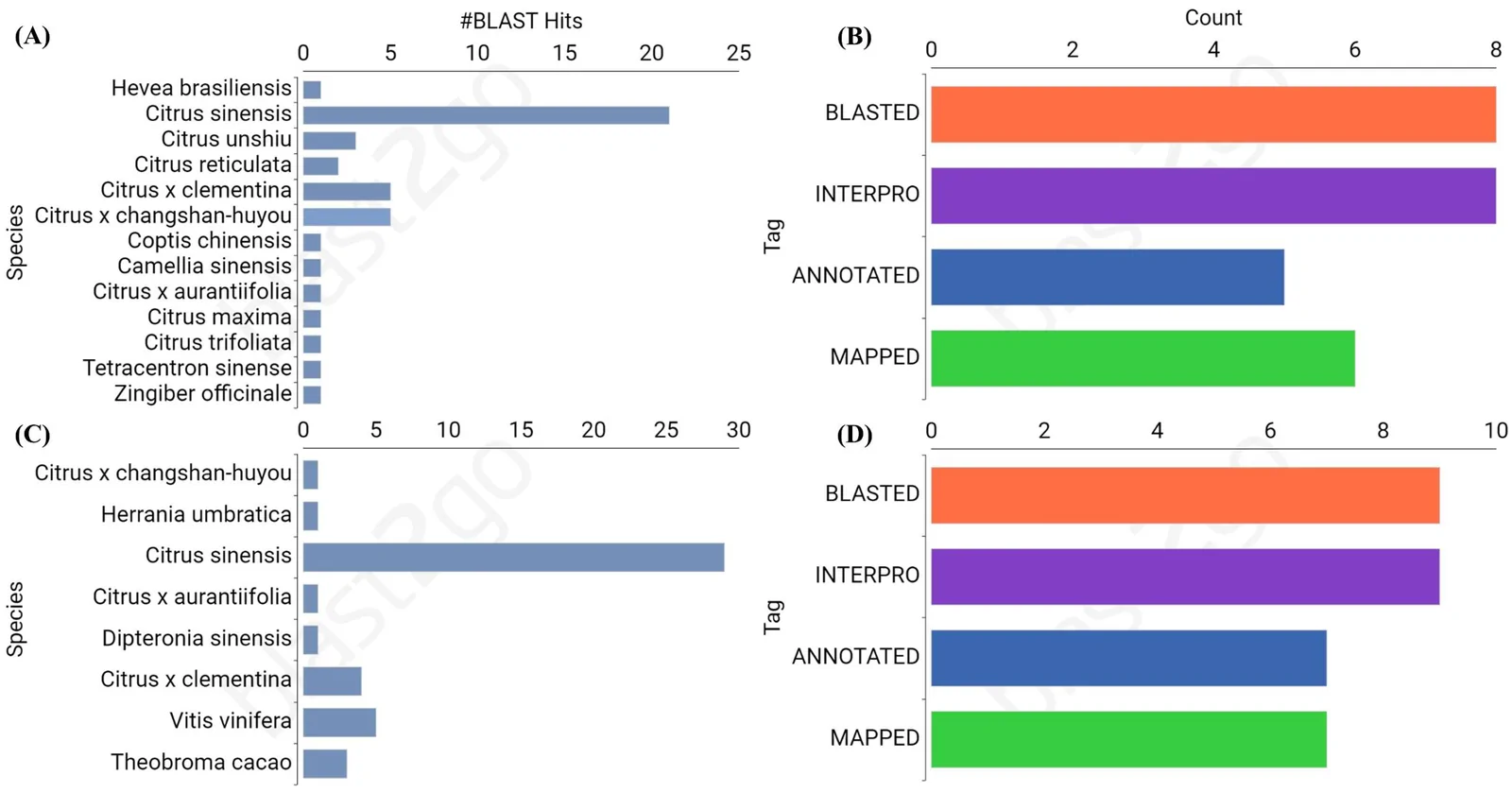
Functional annotation of candidate DArTseq markers. **(A)** distribution of species corresponding to sequences associated with drought tolerance; **(B)** distribution of BLAST hits for markers associated with drought tolerance; **(C)** distribution of species corresponding to sequences associated with vigor reduction and **(D)** distribution of BLAST hits for markers associated with vigor reduction.

The gene sequences obtained from the *C. sinensis* genome were subjected to BLASTx analysis using the Blast2GO software against the NCBI nr database, with a taxonomic filter for Viridiplantae. The sequences were analyzed using InterProScan and generated Gene Ontology (GO) terms during the mapping step (**Table 5** and **Supplementary Table 2**). Most BLAST hits corresponded to *C. sinensis*, followed by other *Citrus* species such as *C. aurantifolia*, *C. unshiu*, and *C. clementina*, with few hits in more distantly related species, as shown in **Figure 4**.

**Table 5:** Summary of the functional annotation of genes putatively related to drought tolerance and vigor reduction in citrandarins.

DArTseq associated marker	Description	Length	e-Value	Sim mean %
Putatively associated with drought tolerance				
100026369|F|0	protein kinase domain-containing protein	12442	0.0	95.29
100019255|F|0	polygalacturonase At1g48100	4547	8,86E-87	99.75
100010748|F|0	putative disease resistance protein RGA3	5928	0.0	99.88
100023347|F|0	Receptor-like protein 33	3234	0.0	99.68
100046832|F|0	S-locus F-box protein	1125	0.0	94.76
100023207|F|0	Uncharacterized vacuolar membrane protein YML018C isoform X1	2096	7,26E-60	53.91
Putatively associated with dwarfing vigor				
100033871|F|0	Agamous-like MADS-box protein AGL6	4186	8,06E-14	91.81
100009554|F|0	ADP-ribosyl cyclase/cyclic ADP-ribose hydrolase	10616	0.0	84.76
100010962|F|0	ADP-ribosyl cyclase/cyclic ADP-ribose hydrolase	13300	0.0	91.75
100010962|F|0	Endonuclease	97709	0.0	96.58
100010962|F|0	hypothetical protein WN944_006731	81207	0.0	96.32
100010962|F|0	ADP-ribosyl cyclase/cyclic ADP-ribose hydrolase	6267	0.0	87.28
100048650|F|0	Retrovirus-related Pol polyprotein from transposon TNT 1-94	26060	0.0	83.3
100053083|F|0	Agamous-like MADS-box protein AGL6	4186	8,06E-14	91.81
100021565|F|0	serine/threonine-protein kinase At3g07070	4976	2,26E-142	70.77

#### 3.4.1 Markers putatively associated with drought tolerance

In general, *C. sunki* has been associated with greater adaptation to water deficit conditions. Studies on *C. sunki* selections have demonstrated their potential for drought tolerance, such as ‘Sunki Tropical’ and ‘Sunki Maravilha’ (Neves et al., 2013; Santana-Vieira et al., 2016; Sousa et al., 2022). Thus, it can be inferred that the markers identified in this study may be associated with genetic mechanisms involved in tolerance to water deficit.

The functional annotation of the gene co-localized with marker 100026369|F|0 revealed an annotation corresponding to a protein kinase. Protein kinases are enzymes that phosphorylate other proteins using ATP. There are several types of kinases that are active at different stages of the cell cycle (Taiz and Zeiger, 2017). Protein phosphorylation/dephosphorylation events are important signaling processes induced by osmotic stress in higher plants. SNF1-related protein kinase 2 (SnRK2), SRK2C, is an osmotic stress-activated protein kinase in *Arabidopsis thaliana* that can significantly impact drought tolerance in *Arabidopsis* plants (Umezawa et al., 2004).

A gene associated with marker 100019255|F|0 showed similarity to polygalacturonase At1g48100. Polygalacturonases belong to the glycosyl hydrolase family and are essential homogalacturonan (HG)-hydrolyzing enzymes involved in several plant developmental processes, such as cell elongation, organ abscission, fruit ripening, microspore release, and pollen tube growth (Hadfield and Bennett, 1998). Polygalacturonases (PGs) modify pectins to regulate cell wall chemistry and mechanics, thereby influencing plant development (Safran et al., 2023). In *Arabidopsis thaliana*, deletion or overexpression of PGX3 (At1g48100) affects both cotyledon shape and the spacing and dimensions of developing stomatal pores. Loss of PGX3 impairs proper stomatal closure, while overexpression accelerates stomatal opening (Rui et al., 2017).

The gene linked to marker 100010748|F|0 was annotated as a putative disease resistance protein RGA3, a gene associated with biotic stress and involved in defense responses against other organisms. Plants possess efficient mechanisms to detect and respond to pathogen infections, such as resistance gene analogs (RGAs), which are considered potential resistance (R) genes. These genes contain conserved domains and features that play specific roles in pathogen defense (Sekhwal et al., 2015). RGA markers have already been used in Citrus for the identification, tagging, and mapping of disease resistance genes in hybrids of *C. unshiu* and *C. nobilis* (Ragayatsu et al., 2014).

Marker 100023347|F|0 was co-localized with a gene annotated as Receptor-like protein 33. Receptor-like proteins (RLPs) are cell surface receptors that play a role in disease resistance in several plant species. In addition, RLPs may be involved in plant development, such as regulating stomatal distribution (TMM) and maintaining the meristem in *Arabidopsis* (Wang et al., 2008).

The gene associated with marker 100046832|F|0 was annotated as S-locus F-box protein. The S-locus controls self-incompatibility and contains S determinants from the pistil and pollen. In Rutaceae, pollen S determinants generally comprise one or more F-box genes closely linked to S-RNase (Liang et al., 2020).

F-box proteins belong to one of the largest protein families in plants and are involved in the regulation of many essential physiological processes, including the growth and development of roots, leaves, stems, flowers, and fruits, as well as responses to biotic and abiotic stresses (Xu et al., 2021). Regarding root development, some proteins from this family (MAX2) can inhibit primary root growth and promote root hair development (Carbonnel et al., 2020). Additionally, through auxin signaling, F-box genes (CEGENDUO) inhibited lateral root formation in *Arabidopsis* (Dong et al., 2006).

Finally, a gene co-localized with marker 100023207|F|0 was annotated as an uncharacterized protein, Uncharacterized vacuolar membrane protein YML018C isoform X1. The YML018C protein from the yeast *Saccharomyces cerevisiae* has not yet had its function determined. High-throughput studies have reported that this protein is localized in the vacuolar membrane (Sturgeon et al., 2021).

Evaluating drought tolerance induced by ‘Rangpur’ lime rootstock in sweet orange (*C. sinensis*), it was possible to verify the transcriptional activation of genes related to soluble sugar accumulation, antioxidant metabolism, cell wall processes, biotic and abiotic stress responses, transcription factors, protein kinases, and abscisic acid (ABA) signaling, as well as the downregulation of genes associated with photosynthetic light reactions, starch metabolism, and ethylene signaling (Gonçalves et al., 2019). These results highlight the complexity of molecular responses associated with adaptation to water deficit in citrus.

Although drought tolerance mechanisms in citrus depend on the maintenance of cellular structures and metabolic processes, it is important to emphasize that the signaling, regulatory, and functional genes that confer drought tolerance are still not fully understood in citrus (Dahro et al., 2023). Our results indicate mechanisms related to stress signaling and perception, as well as cellular adjustment and development.

#### 3.4.2 Markers putatively associated with dwarfing growth habit

The male parental *P. trifoliata* has been associated with the induction of reduced canopy size. It is considered a dwarfing rootstock, as scions grafted onto it exhibit relatively small stature; however, it is more sensitive to drought than most rootstocks (Pompeu Junior, 2005). Thus, the genes annotated in this study may be related to mechanisms involved in the induction of reduced scion growth.

Among the putatively associated markers, markers 100033871|F|0 and 100053083|F|0 were co-localized with a gene annotated as Agamous-like MADS-box protein AGL6.The MADS-box gene family is fundamental to several aspects of plant biology, particularly growth, development, and environmental adaptation, playing a role in the regulation of genes related to flowering, fruit ripening, and stress tolerance (Zhang et al., 2024). The AGAMOUS-like6 (AGL6) gene is a subfamily of MADS-box genes required for floral development in wheat (Kong et al., 2022).

However, recent studies have demonstrated that the functions of this subfamily are more extensive. A meta-analysis of the MADS-box gene family found that some members exert a more indirect effect on root growth and development. In that study, AGL6 was also associated with leaf movement, a process involved in regulating the plant circadian clock, and was found to be expressed in pollen and seeds, indicating a role at different stages of plant development (Adhikari and Kasahara, 2024).

Beyond its role in floral development, MADS-box family genes in *Arabidopsis* have been shown to promote root elongation while also acting as inhibitors of root growth, potentially being involved in root architecture (Castañón-Suárez et al., 2024). In alfalfa (*Medicago sativa*), AGL6, together with SPL12 (SQUAMOSA-PROMOTER BINDING PROTEIN-LIKE), forms a genetic module that regulates root development and nodule formation (Nasrollahi et al., 2022).

Transgenic *Arabidopsis* plants with ectopic expression of OMADS1 (an AGL6 homolog) exhibited significantly reduced plant size, markedly early flowering, and loss of inflorescence indeterminacy. However, these effects were observed under artificial expression conditions, which complicates their interpretation (Hsu et al., 2003).

The genes associated with markers 100009554|F|0 and 100010962|F|0 were annotated with terms related to ADP-ribosyl cyclase/cyclic ADP-ribose hydrolase. This enzyme is involved in the production and degradation of cADPR (cyclic ADP-ribose), a signaling molecule derived from NAD^+^;. cADPR acts as a second messenger, primarily associated with intracellular Ca²^+^; mobilization (Guse, 2000).

Cyclic ADP-ribose (cADPR) was identified as a signaling molecule involved in the response to the hormone abscisic acid (ABA) and was shown to exert its effects through calcium signaling. Experiments demonstrated that cADPR levels in *Arabidopsis* plants increased in response to ABA treatment and prior to ABA-induced gene expression (Wu et al., 1997). The cADPR pathway is important for the regulation of gene expression in plants. Treatment of plant cells with the hormone ABA increased cADPR levels, activating specific genes. Another important function in plants, in which cADPR plays a role, is mediating the activation of genes involved in defense against pathogens (Lee, 2001).

The gene linked to marker 100048650|F|0 was annotated as Retrovirus-related Pol polyprotein from transposon TNT 1-94. Transposable elements, or transposons, represent important components of plant genomes and may contribute to genetic variability and the regulation of gene expression. Transposons, also known as “jumping genes,” have the capacity to insert a copy of themselves into a new location within the genome, moving from one position to another (Taiz and Zeiger, 2017).

A marker (100021565|F|0) was co-localized with a gene annotated as serine/threonine-protein kinase At3g07070 (PBL26). Protein kinases are enzymes that catalyze the phosphorylation of other proteins by adding a phosphate group from ATP, thereby modifying their properties. In plants, kinases act in signal transduction pathways related to growth, development, and responses to environmental stimuli (Taiz and Zeiger, 2017).

This gene may be involved in plant growth and development (Choi and Oh, 2025). In *Arabidopsis*, this kinase plays a crucial role in signal transduction that triggers pollen tube rupture. In angiosperms, the pollen tube enters the receptive synergid cell, where it ruptures to release the sperm cells. These genes encode proteins localized in the plasma membrane, and their deletion causes delayed pollen tube rupture (Xu et al., 2024).

The genetics underlying tree size control in dwarf citrus varieties are still not fully understood. Different physiological mechanisms have been reported, including the involvement of plant hormones such as gibberellins, auxins, brassinosteroids, and abscisic acid, which appear to have a strong relationship with the regulation of this trait (Chu et al., 2025).

In ‘Flying Dragon’ and *P. trifoliata*, a potential mechanism regulating the dwarfing phenotype has been identified, associated with phytohormone signal transduction, sugar and starch degradation, lignin synthesis, and cellulose and hemicellulose degradation processes. In addition, several transcription factors, long non-coding RNAs (lncRNAs), and alternative splicing (AS) events have been identified, which may act as important contributors to the regulation of stem elongation and development in slow-growing rootstocks (Gu et al., 2023). In this study, the genes co-localized with the identified candidate molecular markers were related to protein kinases, MADS-box transcription factors, and cellular signaling.

Although the results obtained allow us to identify possible genomic regions putatively associated with the evaluated characteristics, it is important to emphasize that the associations identified in this study are exploratory in nature, since they are based on correlative evidence. Thus, we highlight candidate genes with biologically plausible functions previously reported in the literature, avoiding excessive generalizations. However, further validation is needed to confirm these associations.

## 4 Conclusion

The rootstocks H248, H70, H151, H139, H124, H18, and W2 induced greater height (≥3.5 m) and larger canopy volume (>30 m³) in ‘Pera’ sweet orange. In contrast, the citrandarin hybrids H110, H68, H137, and H26 produced shorter plants (≤2.8 m) and, consequently, smaller canopy volume (≤20 m³).

The rootstocks H18, H73, H139, H148, H150, H152, H248, H299, and SW exhibited better drought tolerance scores. Conversely, H128, H151, H26, H68, H70, H110, W2, H124, H137, and H47 showed the poorest scores.

Overall, the more vigorous rootstocks displayed greater tolerance to water deficit, whereas the dwarfing rootstocks proved less tolerant. Noteworthy are the citrandarin hybrids H152, H148, H73, and H299, which combined reduced vigor with increased drought tolerance, making them suitable for high-density planting.

The integrated approach of field phenotyping and *in silico* BSA combined with DArTseq markers enabled the detection of 12 molecular markers potentially associated with the agronomic traits of interest. Of these, six were related to dwarfing habit and six to drought tolerance.

Functional annotation of the genes co-localized with the markers revealed candidate genes involved in processes such as stress signaling, hormonal regulation, cellular adjustment, defense responses, and plant development.

The genes associated with drought tolerance are related to stress signaling (protein kinases), cell wall modification (polygalacturonases), disease resistance (RGA3, RLPs), and hormonal regulation (F-box proteins). For the dwarfing trait, the identified genes are linked to developmental regulation (MADS-box transcription factors), calcium signaling (ADP-ribosyl cyclase), and signal transduction (serine/threonine-protein kinases).

These findings point to multigenic and multifactorial control of the phenotype, indicating that drought tolerance and vegetative growth regulation are complex traits, likely governed by more than one gene. However, the marker–trait associations identified here are exploratory in nature and require further validation to confirm the contribution of these genes.

## Data Availability

The original contributions presented in the study are included in the article/**Supplementary Material**, further inquiries can be directed to the corresponding author/s.
